# Borates expand their reduction power

**DOI:** 10.1038/s42004-024-01171-w

**Published:** 2024-04-25

**Authors:** Johannes Kreutzer

**Affiliations:** Nature Communications, https://www.nature.com/ncomms/

**Keywords:** Organometallic chemistry

## Abstract

The chemical reduction of group 1 metal cations to their zero-valent species is challenging. Now, a bipyridine-stabilized borate anion joins the ranks of suitable reducing agents and also proves active in the two-electron reduction of CO_2_.

Since their first report in 1947, tetraphenyl borates have been used in organic synthesis and organometallic chemistry as weakly coordinating ions. While these anions show stability under ambient conditions, their oxidation leads to unstable cationic or radical species prone to decomposing to biphenyl products. Decorating the borate anions with non-innocent ligands improves their stability and equips them with redox-active properties. Now, writing in *Nature Communications*, Lu et al. from the City University of Hong Kong report an example of a stable redox-active borate anion capable of reducing lithium ions (10.1038/s41467-024-46948-8)^1^.

**Figure:**
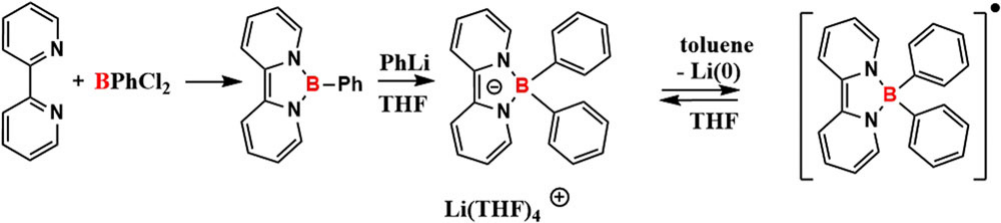
Johannes Kreutzer; Adapted from *Nat. Commun*. 10.1038/s41467-024-46948-8 (2024).

Initially, in search for a masked nucleophilic boryl species, the authors designed a diphenyl borate anion stabilized by bipyridine. Soon it turned out that the B–N bonds were too difficult to cleave for the intended application, but the group quickly also discovered the strong reduction properties of this species.

During the preparation, the authors reacted a known bipyridyl borylene compound with phenyllithium to yield a bipyridine stabilized diphenyl borate anion. The Li ion, which is coordinated by THF, acts as a counter-ion to balance the charge. Replacing the solvent THF for non-coordinating toluene destabilizes the Li ion, which undergoes reduction to its zero valent state. Concomitantly, a stable boron radical is formed. Replacing toluene with THF reverts the reaction, leading to the initial borate anion and the THF stabilized Li counter ion.

The solvent dependence of the reduction reaction shows that the negative potential of the Li ion alone does not dictate whether or not the cation can be reduced. The chemical environment, such as the coordination environment around the ion, is crucial—as also evidenced in another recent *Nature Communications* publication^2^.

Bipyridylboronium compounds are known to undergo two-electron reduction reactions. Testing whether the bipyridyl borate anion shows comparable properties, Lu and team added CuCl to the boron radical, yielding a boronium species.

Encouraged by this two-electron transfer process, which oxidizes the borate anion to the boronium species, the group successfully demonstrated the full potential of the reducing agent in carbon–carbon coupling of pyridine and reduction of organohalides to form the respective P–P, Sn–Sn, Se–Se, and Ge–Ge homocoupling products. Finally, the authors turned their attention to the reduction of CO_2_, reacting the borate potassium species with excess CO_2_ under the formation of K_2_CO_3_ and CO as side-products. “Most surprising to us was the reactivity of the borate anion. It can reduce Li ions to their metallic species, and promotes various coupling reactions and the catalytically two-electron reduction of CO_2_,” summarizes Lu.

“In the future, we will focus on functionalizing the borate anion to see the effects of such modifications on the reactivity,” concludes Lu. “It is also worth further exploring the two-electron-transfer reactivity in organic synthesis and catalysis.”

## References

[1] Li H (2024). Reduction of Li^+^ with a borate anion. Nat. Commun..

[2] Pearce KG (2023). Alkali metal reduction of alkali metal cations. Nat. Commun..

